# Five phases of formamide formed from 0.1 to 1.8 GPa

**DOI:** 10.1007/s11224-026-02744-2

**Published:** 2026-03-30

**Authors:** Alice Dawson, Laura E. Budd, David R. Allan, Richard M. Ibberson, William G. Marshall, Simon Parsons

**Affiliations:** 1 EaStCHEM School of Chemistry and Centre for Science at Extreme Conditions, The University of Edinburgh, King’s Buildings, West Mains Road, Edinburgh, EH9 3FJ UK; 2 ISIS Neutron and Muon Source, Rutherford Appleton Laboratory Harwell Campus, Didcot, Oxfordshire OX11 0QX UK; 3 Present Address: Diamond House, Harwell Science and Innovation Campus, Didcot, Oxfordshire OX11 0DE UK; 4 Present Address: Neutron Technologies Division, Oak Ridge National Laboratory, 1 Bethel Valley Road, Oak Ridge, TN 37831 USA

**Keywords:** Formamide, High-pressure crystal structures, Polymorphism, Pixel calculations, Topology

## Abstract

Formamide, the simplest amide, forms an ambient-pressure crystal structure consisting of stacks of NH⋯O hydrogen-bonded layers. Here we report the effect of pressure extending to a maximum of 3.6 GPa on the crystal structures of formamide-*h*_3_ and formamide-*d*_3_ using single-crystal X-ray diffraction and neutron powder diffraction, revealing previously unrecognised but extensive phase diversity between ambient pressure and 1.81 GPa. The crystal structures of five distinct crystalline phases, designated I_α_ (the known ambient pressure phase), II, III, IV_β_ (also known from previous work) and V, have been determined. The pressures and isotopologues of the determinations were 0.20 GPa (for *h*_3_ phase I_α_), 0.20 and 0.36 GPa (for the *d*_3_ and *h*_3_ forms of phase II), 0.35 GPa (*d*_3_ form of phase III), 0.43 and 0.78 GPa (for the *h*_3_ and *d*_3_ forms of IV_β_) and 1.81 GPa (*d*_3_ form of phase V). All phases contain NH⋯O hydrogen-bonded chain motifs, with phases III and IV_β_ being layered structures similar to I_α_, but phases II and V forming 3-dimensional networks. The architectures are determined by whether a hydrogen-bonded chain utilises the oxygen lone pair cis or trans to the amide nitrogen and the orientations of the molecules. The phases conform to well-known topological types, I_α_, II and IV_β_ being based on cubic close packing while phase V is a distorted form of the same topology, reflecting denser packing at elevated pressure. Phase III can be considered to have a body centred cubic topology. Pixel packing energy calculations show that competing H-bonding arrangements are energetically comparable even when they deviate from an ideal H-bonding geometry, while non-H-bonding contacts, including antiparallel stacking and aldehyde–aldehyde interactions, contribute additional stabilisation, also with notable geometric flexibility.

**Graphical Abstract:**

**Figure:**
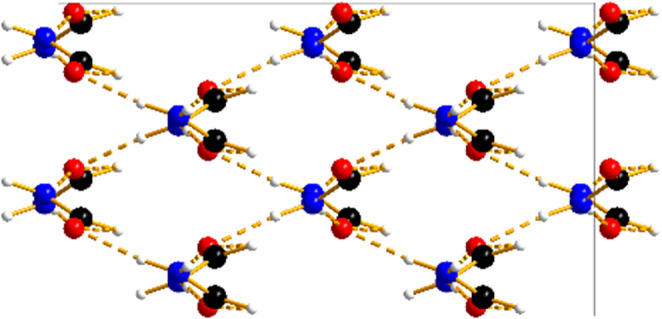
The simplest amide, formamide, forms no less than five different phases between ambient pressure and about 2 GPa.

**Supplementary Information:**

The online version contains supplementary material available at 10.1007/s11224-026-02744-2.

## Introduction

Formamide is the simplest amide and the simplest molecule containing the CONH moiety of the peptide bond. The crystal structure was first determined by Ladell & Post in 1954 at 223 K using X-ray single-crystal diffraction [1]. An electron density study at 90 K [2] and neutron diffraction at 10 K [3] found the same monoclinic structure in *P*2_1_/*n*, consisting of NH⋯O hydrogen-bonded chains of molecules with further hydrogen bonds linking the chains into layers. There are no hydrogen-bonding interactions between the layers. We shall refer to this as the I_α_ phase.

Raman spectroscopy has been used to study the behaviour of formamide at low temperatures [3] and at high pressure [4]. The high pressure study indicated a phase transition at 5.0 GPa, identified by a change in the pressure dependence of the intramolecular and intermolecular vibrational modes. There has been one crystallographic high-pressure study by Katrusiak and co-workers [5] in which a new β phase was formed on high-pressure crystallisation from the liquid. Data were collected at 0.88 and 1.20 GPa. More details on this study are given below.

High pressure is a powerful means for exploring the energy landscape of molecular materials [6], and in this paper we describe the effect of pressure on the crystal structure of formamide in both its isotopically normal and perdeuterated forms using both single-crystal X-ray and neutron powder diffraction. We show that it exhibits remarkable polymorphism at ambient temperature and pressures between 0.1 GPa and 1.8 GPa, forming no less than five phases, in marked contrast to the one structure known under varying conditions of temperature from 20 K up to the melting point (275.7 K).

## Experimental

### X-ray single-crystal diffraction

A sample of formamide (systematic name methanamide, Aldrich, used as received) was loaded into a Merrill-Bassett diamond anvil cell equipped with 600 μm diamonds and a tungsten gasket [7]. A small ruby chip was also loaded into the cell as a pressure calibrant and the ruby fluorescence method used to measure the pressure [8]. Application of pressure caused the sample to crystallise into a polycrystalline mass. This was then heated until the crystallites began to melt. Heating was stopped when only one crystallite remained. The crystallite grew to fill the cavity as the cell was allowed to cool back to room temperature.

Several crystals were grown using this method at various pressures. Experiments showed that the low temperature phase (formamide I_α_) could be grown at 0.20 GPa while two different phases could be grown at 0.36 GPa and 0.44 GPa, which we will refer to as phases II and IV_β_, respectively, phase nomenclature being justified in the first Section of the Results and Discussion.

Diffraction data were collected with Mo-K_α_ radiation using a Bruker SMART Apex diffractometer. Data collection and processing were as described by Dawson et al. [9]. Integrations were carried out using the program Saint [10] and absorption corrections with Sadabs [11]. Data were merged in Sortav [12].

Refinement of phase I_α_ at 0.2 GPa used the previously determined coordinates (CSD [13] refcode FORMAM02) [2] as a starting point. The structure was refined against |*F*|^2^ using Crystals [14]. The two high pressure structures were solved using direct methods (Shelxs) [15] and also refined against |*F*|^2^ using Crystals. Non-H atoms in phases I_α_ and IV_β_ were refined with anisotropic displacement parameters subject to rigid bond restraints; those in phase II were refined isotropically as completeness was relatively low. The CO and CN distances and the OCN angle were restrained to 1.24(1) Å, 1.32(1) Å and 125(1)° in the refinements of phases I_α_ and II; in IV_β_ the two molecules composing the asymmetric unit were restrained to be geometrically similar. All hydrogen atoms were placed geometrically and refined subject to geometric restraints. Crystal and refinement data are given in Table 1.

### Neutron powder diffraction

High pressure neutron powder diffraction data were collected using the time-of-flight technique on the PEARL instrument at ISIS Neutron and Muon Source [16]. Formamide-*d*_3_ (CDN isotopes, used as received) was contained in a null-scattering Ti-Zr alloy capsule gasket and loaded into a Paris-Edinburgh cell. The sample was loaded with powdered silica wool to facilitate formation of a randomly orientated powder. A small pellet of lead was also loaded as a pressure marker [17]. The Paris-Edinburgh cell ram pressure was monitored and controlled by a computer controlled hydraulic system.

Upon application of pressure, the sample crystallised at 0.54 GPa in phase IV_β_. The pressure was increased in small steps from 0.54 GPa to a final pressure of 3.6 GPa. On increasing the pressure from 1.3 GPa to 1.83 GPa, the sample underwent a phase transition to a previously unobserved phase V. No further phase transitions were observed on increasing the pressure. By 3.6 GPa, peak broadening was fairly pronounced, and the sample was decompressed. During the decompression the sample transformed back to phase IV_β_ at 1.15 GPa and then to phase II at 0.13 GPa. Further decompression resulted in the sample melting.

**Fig. 1:**
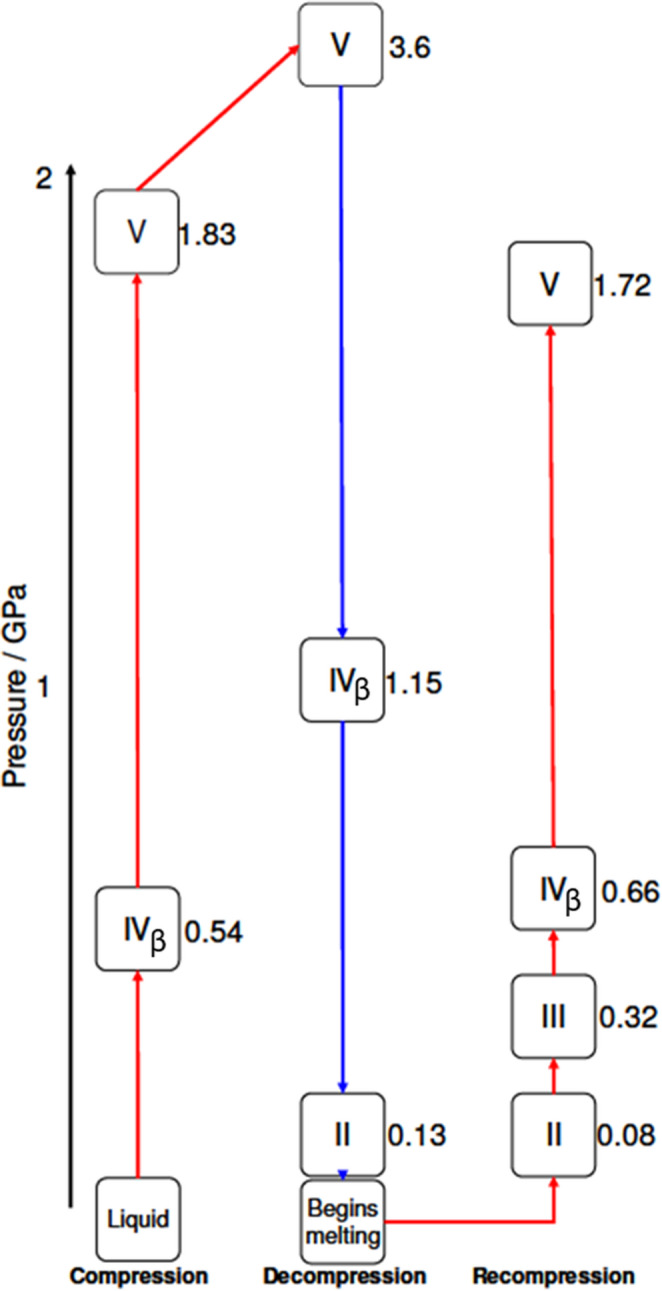
Sequence of high-pressure phase transitions observed by neutron diffraction for formamide-*d*_3_. Pressures are given in GPa

Recompressing the sample led to formation of phase II at 0.08 GPa. Further compression from 0.08 GPa to 0.32 GPa resulted in formation of another previously uncharacterised phase, phase III. Further increasing the pressure resulted in transitions to phase IV_β_ at 0.66 GPa and then phase V at 1.72 GPa. The sequence of compression, decompression and recompression described above is summarised in Fig. 1.

In order to obtain high quality diffraction patterns of the new phases for indexing and structure solution, a second sample was loaded into the Paris-Edinburgh cell without the lead pressure calibrant present. The sample initially crystallised in phase IV_β_ at 0.78 GPa before transforming to phase V on increasing the pressure. While decompressing from 2.26 GPa the sample transformed down through all the high pressure phases, finishing in phase II. The structures were refined using data collected at 0.2 GPa (phase II), 0.35 GPa (phase III), 0.78 GPa (phase IV_β_) and 1.81 GPa (phase V). In order to obtain the pressures quoted for the final structures in the absence of a pressure calibrant, the data collected earlier in the experiment with the lead calibrant present were used to plot the unit cell volume as a function of the pressure for each phase. For phases IV_β_ and V, a second order polynomial fit was calculated which was then used to obtain the pressure from the unit cell volume. As phases II and III only exist over a very narrow pressure range there were insufficient data to perform a polynomial fit and so the pressures can only be estimated. For phase III a straight line fit was calculated for three data points as the unit cell volume was within the range previously collected with a pressure calibrant. The pressure has been estimated based on this fit. For phase II the unit cell volume was outside the range previously collected with a pressure calibrant, thus it is only possible to estimate a pressure range.

Formamide-I_α_ was not observed in any of the neutron powder investigations.

### Analysis of neutron powder data

**Fig. 2:**
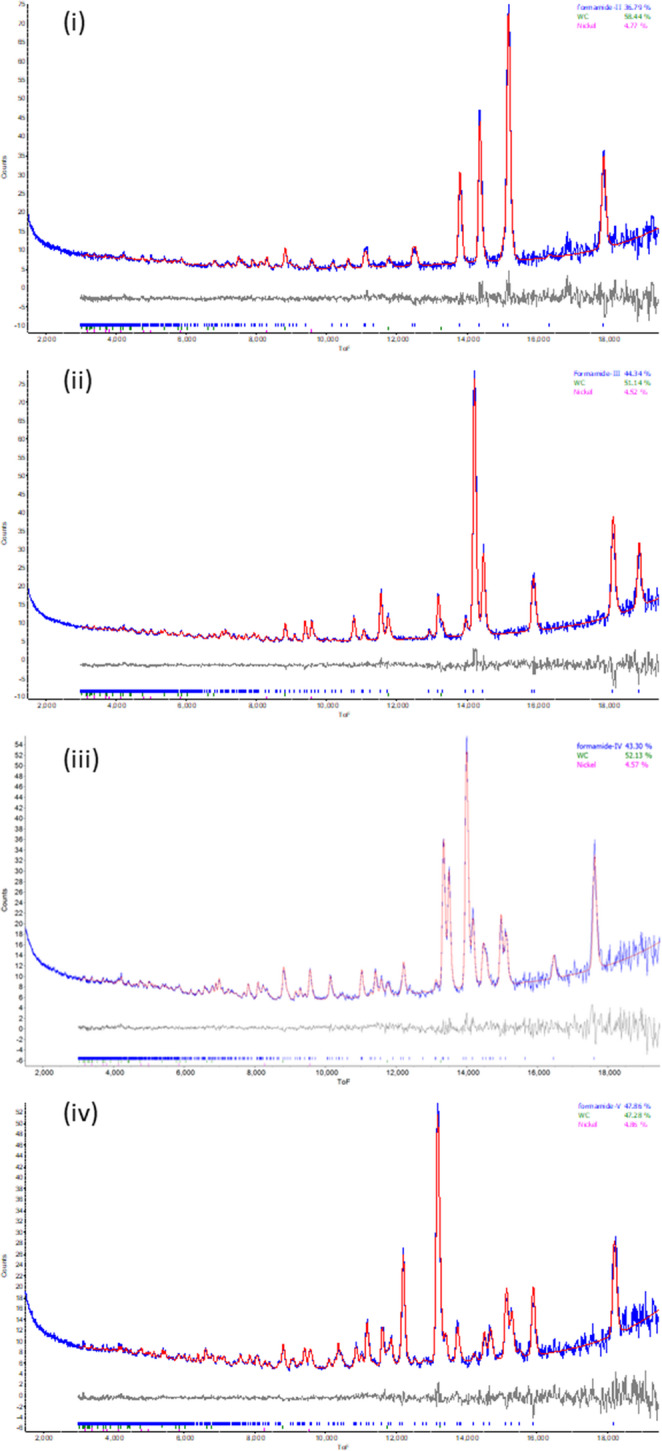
Rietveld fits against neutron diffraction data collected for formamide-*d*_3_ (i) phase II, (ii) phase III, (iii) phase IV_β_ and (iv) phase V

The powder patterns of phases III and V were indexed with DICVOL91 [18] and solved by simulated annealing in TOPAS-Academic [19]. Rietveld refinements were carried out using TOPAS-Academic. The formamide molecules were treated as planar rigid groups. Bond lengths and angles were taken from X-ray single-crystal data [2], with deuterium distances set to those obtained from an MP2/6-31G** geometry optimisation (Gaussian09) [20] of the isolated molecule (N-H 1.009 Å, C-H 1.050 Å). Bond lengths, angles and torsion angles were not refined. Separate isotropic displacement parameters were refined for the non-deuterium and deuterium atoms. A six-term Chebychev polynomial was used for the background and the peak shapes were modelled with back-to-back exponential functions. As the sample was crystallised in situ, a preferred orientation correction was included. Nickel and tungsten carbide, which arise from the anvils of the Paris-Edinburgh cell, were also included in the refinement. When lead was present in the sample, the unit cell volume was used to estimate the pressure using a Birch-Murnaghan equation of state [17]. Rietveld refinement profiles are shown in Fig. 2 with crystal and refinement data given in Table 2. The cifs were generated using the pdCIF macros described in ref [21].

### Pixel, topological and other calculations

The final crystal structures obtained from the neutron data were used to calculate the molecular electron density for each phase. The single-crystal structure obtained for formamide-*h*_3_ at 0.2 GPa was used for phase I_α_ with H-atom distances reset to standard neutron values. Standard quantum mechanical methods using Gaussian09 at the B3LYP/6-31G** level of theory and basis set were used. Lattice energy calculations were performed using the Pixel method [22] as implemented in the program Pixelc through the MrPixel interface [23] using a cluster of molecules of radius 14 Å and condensation level 4. These calculations yielded a total lattice energy and a breakdown into the electrostatic, polarisation, dispersion and repulsion components. The first coordination sphere of a molecule in a crystal structure is defined here as being composed of molecules making contacts in which the Pauli repulsion term from the Pixel calculations is greater than zero.

The lattice energies obtained from the Pixel calculations are given in Table 3. The values are obtained at different pressures and so are not directly comparable. However, the similarity of the total energies obtained validates the structure solutions obtained from powder data for phases III and V. Tables S1-S5b in the ESI give the energies of the molecule-molecule interactions in the first coordination sphere of each phase.

Topological analyses were carried out using Topos [24] and are based on simplified networks defined using molecular geometric centroids [25]. Care was taken to ensure that the topological calculations were based on the same molecular coordination sphere as identified in the Pixel calculations. In some cases, one or two more molecules were identified by Topos, but these contacts always corresponded to solid angles in the smoothed molecular Voronoi-Dirichlet polyhedron of ~ 0.1 sr (Ω < ~1%) or less. These contacts were manually removed from the adjacency matrix.

Geometrical analyses were carried out in Platon [26] and structures were visualised in Mercury [27].

**Table 1:** Crystal and refinement data for X-ray single-crystal structures for formamide-*h*_3_ phases I_α_, II and IV_β_. All data were collected at 298 K using ω scans on a Bruker Apex diffractometer

Pressure/GPa	Formamide-I_α_	Formamide-II	Formamide-IV_β_
	0.20	0.36	0.43
Chemical formula	CHONH_2_	CHONH_2_	CHONH_2_
*M*r	45.04	45.04	45.04
Cell setting, space group	Monoclinic, *P*2_1_/*n*	Orthorhombic, *Pna*2_1_	Monoclinic, *P*2_1_/*n*
*a*, *b*, *c*/Å	3.6812(4), 9.2666(9), 6.7968(16)	9.4564(11), 3.6639(7) 6.4268(18)	3.6006(6), 18.828(3), 6.3072(9)
α, β, γ/°	90, 99.448(6), 90	90, 90, 90	90, 93.556(11), 90
*V*/Å^3^	228.71(6)	222.67(8)	426.76(11)
*Z*	4	4	8
*D*_calc_ /Mg m^− 3^	1.308	1.343	1.402
Crystal description, size/mm	colourless block 0.3 × 0.3 × 0.15	colourless block 0.2 × 0.2 × 0.1	colourless block 0.3 × 0.3 × 0.15
Data Collection			
Absorption correction	Multiscan	Multiscan	Multiscan
* T*_min_	0.55	0.37	0.53
* T*_max_	0.98	1.00	0.98
No. of measured, independent and observed reflections	720, 165, 158	460, 113, 111	3427, 445, 439
Criterion for observed reflections	*I* > 2 σ (*I*)	*I* > 2 σ (*I*)	*I* > 2 σ (*I*)
* R*_int_	0.025	0.046	0.0611
θ_max_ (°)	26.54	23.19	25.1
Refinement			
Refinement on	*|F|* ^2^	*|F|* ^2^	*|F|* ^2^
* R*[*F*^2^ > 2σ(*F*^2^)], *wR*(*F*^2^), *S*	0.057, 0.155, 1.19	0.138, 0.251, 1.15	0.067, 0.133, 1.14
No. of parameters	28	25	80
Δρ_max_, Δρ_min_ (e Å^–3^)	0.08, − 0.12	0.34, −0.34	0.22, − 0.20

**Table 2:** Crystallographic and refinement data for formamide-*d*_3_ phases II to V as determined by neutron powder diffraction. All data were collected at 298 K using the time-of-flight method on the PEARL instrument at ISIS

Phase	Formamide-II	Formamide-III	Formamide-IV_β_	Formamide-V
Pressure/GPa	0.20	0.35	0.78	1.81
Chemical formula	CDOND_2_	CDOND_2_	CDOND_2_	CDOND_2_
*M*_r_	48.05	48.05	48.05	48.05
Cell setting, space group	Orthorhombic, *Pna*2_1_	Monoclinic, *P*2_1_/*a*	Monoclinic, *P*2_1_/*n*	Monoclinic, *P*2_1_/*a*
*a*, *b*, *c* /Å	9.498(2), 3.7509(3), 6.4195(14)	6.9395(4), 6.7987(7), 5.3021(4)	3.5850(2), 18.8144(17), 6.2741(7)	12.6650(31), 3.4694(2), 10.1897(9)
α, β, γ/°	90, 90, 90	90, 117.147(6), 90	90, 93.645(6), 90	90, 119.743(8), 90
*V*/Å^3^	228.70(7)	222.59(3)	422.32(6)	388.75(7)
*Z*	4	4	8	8
*D*_calc_/g cm^− 3^	1.396	1.434	1.512	1.642
Range of *d*/Å	0.64–4.14	0.64–4.14	0.64–4.14	0.64–4.14
Refinement				
Method	Rietveld	Rietveld	Rietveld	Rietveld
*R*_wp_	0.0496	0.0337	0.0312	0.0319
*S*	1.212	1.268	1.243	1.274
Number of parameters	28	31	37	37

**Table 3:** Lattice energies of phases of formamide as calculated using the Pixel method. The energy terms *E* give the electrostatic, polarisation, dispersion, Pauli repulsion and total energies in kJ mol^− 1^. The cell dipole term for phase II is − 1.2 kJ mol^− 1^. The experimental, ambient pressure sublimation energy is 71.9 kJ mol^− 1^, averaged over three determinations

Phase	Pressure/GPa	E_elec_	E_pol_	E_disp_	E_rep_	E_tot_
I_α_	0.20	−81.2	−26.0	−38.7	70.4	−75.6
II	0.20	−78.7	−27.8	−39.7	78.4	−69.0
III	0.35	−77.2	−26.6	−41.3	73.6	−71.4
IV_β_	0.78	−78.8	−26.7	−45.7	79.4	−71.8
V	1.18	−84.4	−28.4	−53.6	97.5	−68.9

## Results and discussion

### Phases of formamide at high pressure

The effect of pressure on the crystal structure of formamide was first investigated by Katrusiak and co-workers, who showed that, both in a piston cylinder press and a diamond anvil cell, the freezing pressure of formamide is between 0.43 and 0.445 GPa at 296 K [5]. They compressed formamide in a diamond anvil cell until it solidified into a polycrystalline mass, which was then melted back until one crystallite remained. A single crystal of formamide grew to fill the cell as it was allowed to cool. A new form was obtained, which they designated the β phase to distinguish it from what they referred to as the α phase, which had been known since the ambient pressure/low-temperature structure was reported in 1954 [1].

Our own work based on single-crystal diffraction measurements followed a very similar route to that described by Katrusiak, but the results are somewhat different. A crystal of the ambient-pressure α form was obtained on solidifying the sample at 0.2 GPa. A single crystal of a new phase II was obtained at 0.3 GPa, while Katrusiak’s β phase formed, as expected, at 0.4 GPa. All of these pressures are somewhat below the freezing pressure quoted above from Katrusiak’s paper. The precision of ruby fluorescence pressure measurements is 0.05 GPa and, while the distinctions between our and Katrusiak’s values are barely statistically significant, there may also be a temperature effect: an ambient temperature of 296 K quoted above would be an unprecedented luxury in our lab in Edinburgh. Formamide may also suffer from the pressure equivalent of supercooling. Finally, it may simply be a matter of differing ruby fluorescence calibrations. The important conclusion, though, is that formamide appears to display greater phase diversity around its freezing pressure than has hitherto been recognised, a finding which is supported by neutron powder diffraction measurements described below.

High pressure neutron powder diffraction experiments have shown formamide-*d*_3_ forms four high pressure phases, phase II at 0.08 GPa, a new phase III at 0.32 GPa, the β phase at 0.66 GPa and another new phase, V, at 1.72 GPa. The low-temperature α phase was not observed in the neutron powder study at all. Sequential formation of all the phases from II to V was shown on compression of phase II and the reverse when decompressing a pure sample of phase V from 2.26 GPa. Decreasing the pressure further results in the sample melting. Note that the formation pressures of phase II and III are also lower than the freezing pressures quoted in Katrusiak’s paper, but deuteration effects may also be at play.

In order to retain a phase nomenclature which follows a simple numerical sequence with increasing pressure we shall refer to the phases described above as I_α_ for the low temperature, ambient pressure phase, II, III, IV_β_ for Katrusiak’s β phase and V.

### Formamide-I_α_

**Scheme 1:**
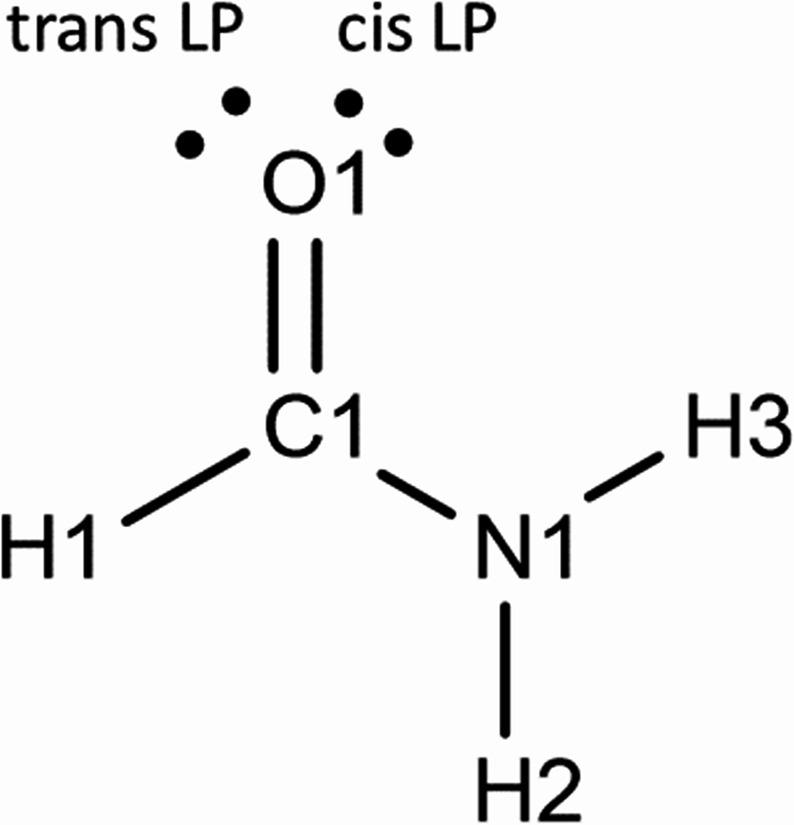
The chemical structure of formamide, showing atom numbering and positions of O-based lone pairs (LP)

The structure of formamide-I_α_ at low temperatures has previously been characterised by both X-ray and neutron diffraction. A single crystal of formamide-I_α_ was obtained using an isotopically normal sample crystallised in a diamond anvil cell at 0.2 GPa. The space group is *P*2_1_/*n* and *Z*’ = 1. Atom numbering used throughout this study is shown in Scheme 1.

**Fig. 3:**
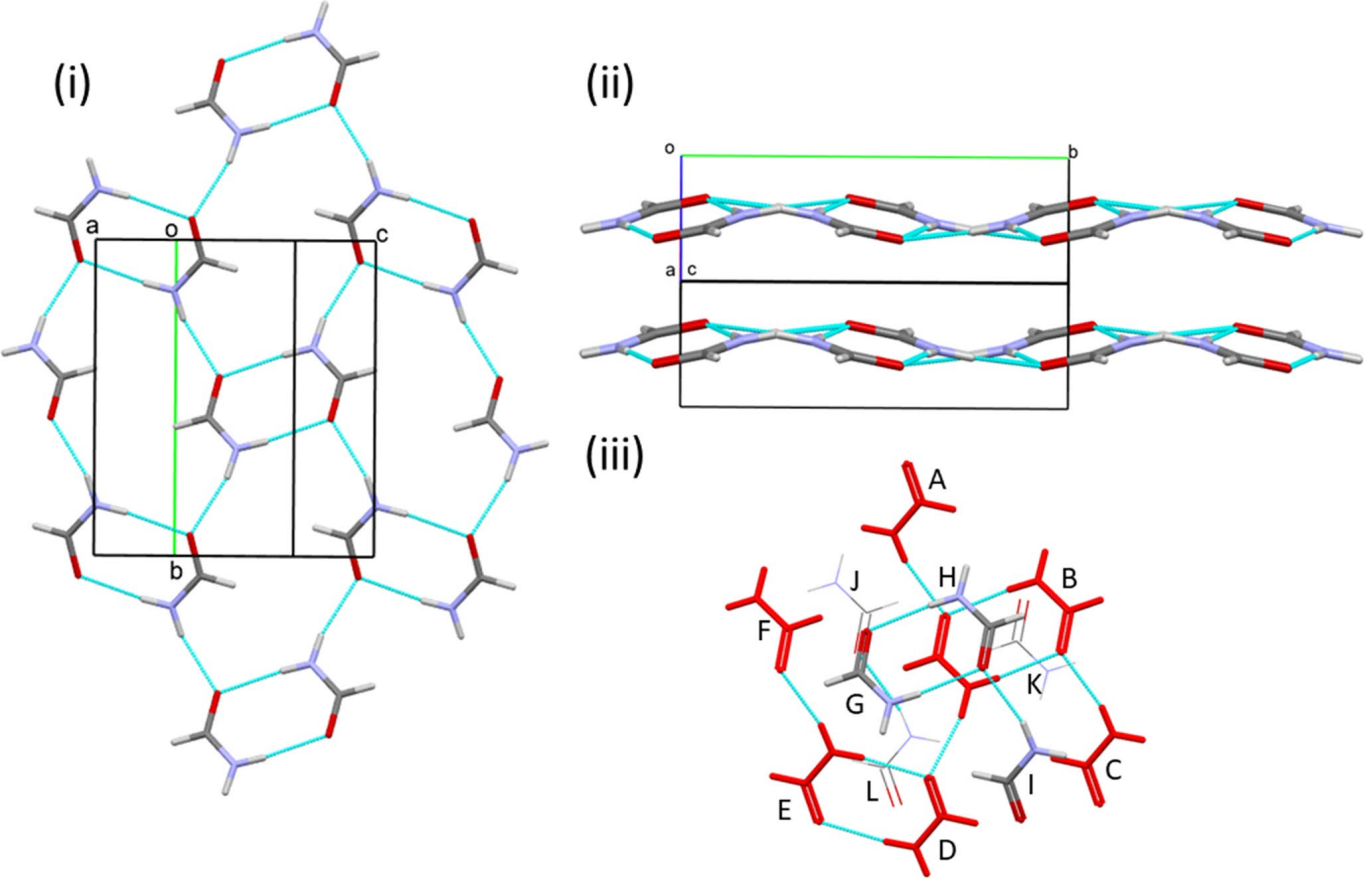
Formamide-I_α_ at 0.2 GPa. (i) Formation of H-bonded layers perpendicular to (101). (ii) Stacking of H-bonded layers. (iii) The molecular coordination sphere. The central layer is shown in red, the lower and upper layers are shown in wireframe and stick format, respectively. The labelling should be compared with Scheme 2i

The structure at 0.2 GPa consists of N1H2⋯O1 H-bonded chains (normalised O1⋯H2 1.89 Å) of formamide molecules parallel to the *b* axis (Fig. 3i). Successive molecules along the H-bonded chain are generated by .2_1_. operations. The angle between the planes of successive molecules is 26.8°. In graph set notation, the chains form a C(4) primary level motif [28].

The formally sp^2^ hybridised oxygen atom has two lone pairs located in the plane of the molecule, in positions which are cis and trans to the amide nitrogen N1 (Scheme 1). The N1H2⋯O1 hydrogen H-bond involves the oxygen lone pair which is trans to N1, leaving H3 and the second lone pair on the same side of the molecule. These connect to other chains across inversion centres to generate R^2^_2_(8) dimers through N1H3⋯O1 hydrogen bonds (O1⋯H3 1.94 Å). The positions of the H-bonding functions is in excellent agreement with a full interaction map [29] (Fig. S1i in the ESI), which depicts the distributions of H-bond donors and acceptors about amides in the Cambridge Structural Database [13].

The combination of the relatively planar, primary level C(4) and R^2^_2_(8) motifs generates secondary level R^4^_6_(16) rings which alternate with the R^2^_2_(8) dimers between pairs of chains. These motifs generate layers parallel to (101), Fig. 3ii; although the molecules do not lie precisely in the layers, the deviations are small, at most 0.505 Å for O1.

The lengths of the hydrogen bonds are not significantly different to those at low temperature. Pixel calculations show the strongest interaction to be the R^2^_2_(8) dimer interaction. The total molecule-molecule energy is − 66.6 kJ mol^− 1^, the magnitude reflecting the formation of two NH⋯O hydrogen bonds within the interaction. The second strongest molecule-molecule interactions are those connected by the N1H2⋯O1 hydrogen bonds to neighbouring molecules along the chain (− 30.1 kJ mol^− 1^). Other interactions within the layers are much smaller in magnitude and are formed across the R^4^_6_(16) rings. Both are long-range electrostatic interactions. The energy of one is − 8.4 kJ mol^− 1^, while the other is weakly destabilising (+ 2.0 kJ mol^− 1^) as a result of juxtaposition of positively charged H-atoms at distances of ~ 3 Å.

**Scheme 2:**
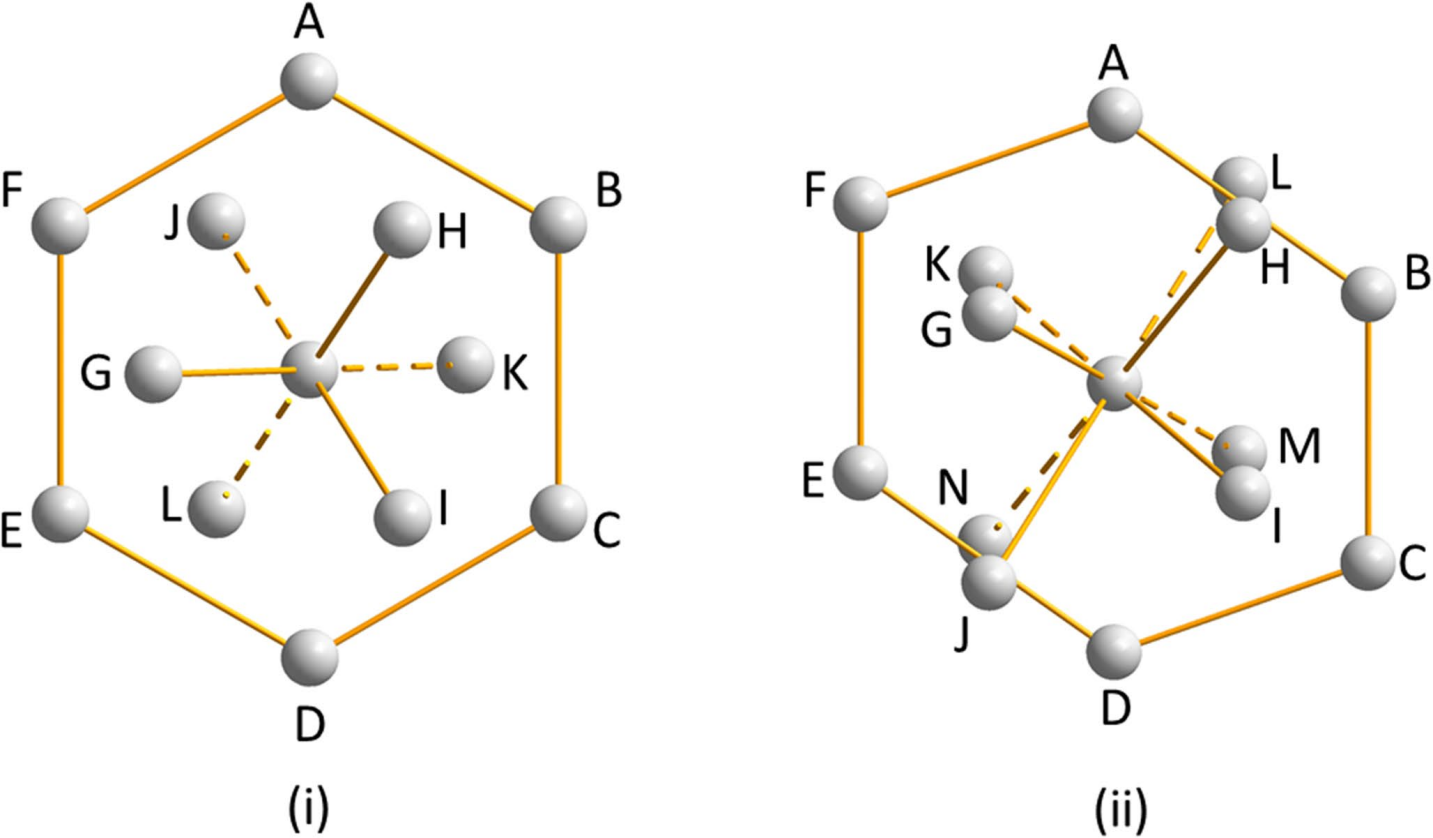
Ideal cubic close packed (i) and body-centred cubic (ii) topologies as observed in hard sphere structures such as Pt and W, respectively. The images show a central layer in which a central reference point is surrounded by six others labelled A-F. In (i) the layers above (connected by solid lines) contains points G-I and the layer below (dashed lines) molecules J-L. The total coordination number is 12. The arrangement in (ii) is similar except that the upper layer contains points G-J, the lower layer points K-N and the coordination number is 14

Within the layers, each molecule is surrounded by six others. The molecular coordination sphere is completed by three molecules in the layer above and three in the layer below in a topologically cubic close packed arrangement (Fig. 3iii and Scheme 2i). In the following discussion, molecular coordination spheres will be depicted with the C(4) chains oriented vertically and forming part of the central layer of the molecular coordination sphere; molecules A-F are located in this layer with molecules in the A and D positions forming the C(4) chain. In the 12-fold molecular coordination of phase I_α_, the sets of three molecules above and below the central layer of the molecular coordination sphere are labelled G-I and J-L, respectively, as shown in Scheme 2i. They are in a staggered arrangement: had they been eclipsed, the topology would have conformed to hexagonal close packing.

A coordination sequence specifies the number of molecules in the first, second, third… molecular coordination spheres. In formamide-I_α_ the coordination sequence is 12-42-92, which is the same as for perfect cubic close packing. Although the ideal symmetry of a hard-sphere cubic close packed arrangement is disrupted by the shape and interactions made by the formamide molecule, the topology nevertheless conforms closely to this arrangement. This topological approach will prove useful in comparing the phases of formamide described here.

There are no H-bonds between the layers, and the interactions take the form of an aldehyde-aldehyde electrostatic interaction formed across an inversion centre with an O1⋯H1 distance measuring 2.62 Å to molecule J and an offset antiparallel stacking interaction to the other layer (stacking distance 3.36 Å) to molecule H. The molecule-molecule energies of these interactions are similar (− 16.9 and − 14.5 kJ mol^− 1^, respectively). Two pairs of symmetry-related interactions are formed to the layers above and below in positions I/L and G/K, one is stabilising (− 3.8 kJ mol^− 1^). The other is generated by a lattice translation along **a** and features the shortest centroid-centroid interlayer distance (3.681 Å), but is destabilising (5.6 kJ mol^− 1^, with an electrostatic component of 10.1 kJ mol^− 1^). The interlayer separation is 3.030 Å.

### Formamide-II

Formamide-II is orthorhombic, space group *Pna*2_1_ with one molecule in the asymmetric unit. The crystal structure was determined using single-crystal X-ray methods at 0.35 GPa and neutron powder diffraction at 0.20 GPa. The parameters from the latter are discussed here.

**Fig. 4:**
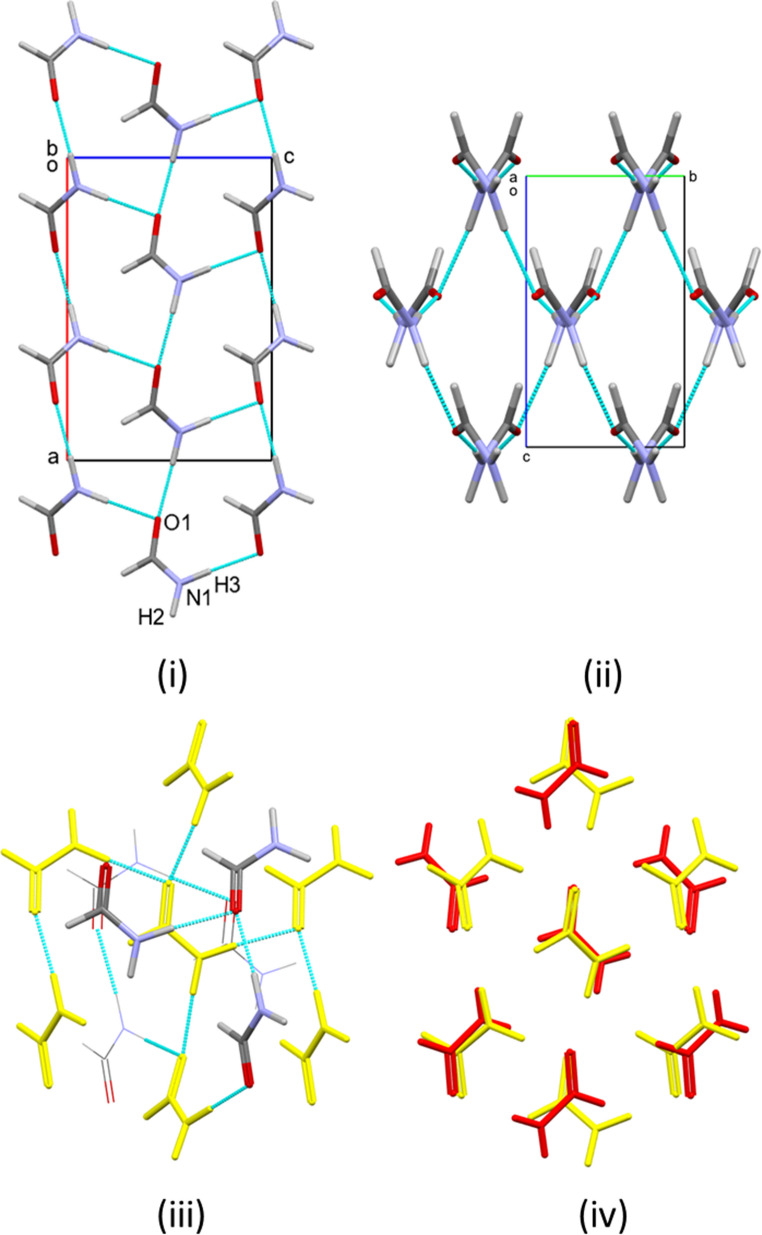
Formamide-II at 0.2 GPa. (i) Projection along **b**. (ii) Projection along **a** showing the 3D network character of the structure. The apparent layers in (i) run diagonally in (ii) with H-bonded links between them. (iii) Coordination sphere, for labelling see Fig. 3iii or Scheme 2i. The central layer is in yellow. (iv) Fit of the O positions in the central layers of the coordination spheres of phases I_α_ (red) and II (yellow)

As in formamide-I_α_, the structure consists of N1H2⋯O1 [O1⋯H2 1.90(2) Å] hydrogen-bonded chains, generating a C(4) motif, Fig. 4i. However, the H-bond now involves the lone pair on O1 which is approximately cis to N1. This change results in the remaining hydrogen bond donor and acceptor being on opposite sides of the molecule, and the R^2^_2_(8) dimer units which characterised phase I_α_ cannot be formed. Instead, N1-H3⋯O1 H-bonds [O1⋯H3 1.881(18) Å] connect to chains on either side. The angle between the planes of successive molecules in the C(4) chains is 54.4° and alternate formamide molecules link to different pairs of chains, building up a three-dimensional network rather than a layered structure (Fig. 4ii). Phase II is the only form of formamide reported here which does not feature R^2^_2_(8) dimer units.

The full interaction map calculated for H-bonding in phase II (Fig. S1ii) shows that the donor functions are quite markedly misaligned with the directions defined by the lines between the maxima and O1. The same misalignment is evident in the angles C1-O1⋯H2 (154°) and C1-O1⋯H3 (105°) which deviate from the expected value of 120° for the optimal interaction with the lone pairs on sp^2^ hybridised oxygen. The molecule-molecule energy of the dimer involving the N1H2⋯O2 H-bond is a little less than it was in phase I_α_ (− 26.7 versus − 30.1 kJ mol^− 1^), while that involving N1H3⋯O1 is, at − 35.7 kJ mol^− 1^, stronger than half the energy of the R^2^_2_(8) dimer in phase I. The quite small magnitudes of the differences and the degree of energetic compensation attest to the geometrical flexibility of the H-bonds in formamide.

Although the numerical values of the unit cell axis lengths are similar to those of phase I_α_, there is no group-subgroup relationship between the structures. The description of the packing in the previous paragraph also makes the structures appear quite different. Nevertheless, phase I_α_ and II are topologically very closely related. In both cases, the molecular coordination sphere contains 12 molecules (Fig. 4iii) with a cubic close packed topology and a coordination sequence of 12-42-92. An overlay based on matching positions of the oxygen atoms in the central layers of the coordination spheres of phases I_α_ and II (Fig. 4iv) shows that the positions of the molecules in the two phases are quite consistent (root mean square difference in O positions *RMS*_O_ = 0.820 Å) and the distinction between the phases arises through molecular reorientations.

The energies of the contacts made to non-H bonded molecules in equivalent positions in the coordination sphere are remarkably consistent in phases I_α_ and II. The most stabilising, with energies of − 12.3 kJ mol^− 1^ are interactions between the OCNH side of one molecule and the aldehyde moiety of another with O⋯H distances of 2.96(2) Å in positions H and J. Similarly, there are two interactions generated by lattice translations (this time, along **b**) which are slightly destabilising in positions G and K, with the sphere being completed by one non-specific dispersion interaction in position I and a slightly destabilising electrostatic interaction in position L, both quite neutral in terms of their contributions to the stabilisation of the structure. The same is true of the non-H-bonded interactions at positions C and E in the central layer of the coordination sphere.

### Formamide-III

The structure of formamide-III is monoclinic, space group *P*2_1_/*a* with *Z*’ = 1. The structure was determined at 0.35 GPa using neutron powder diffraction.

**Fig. 5:**
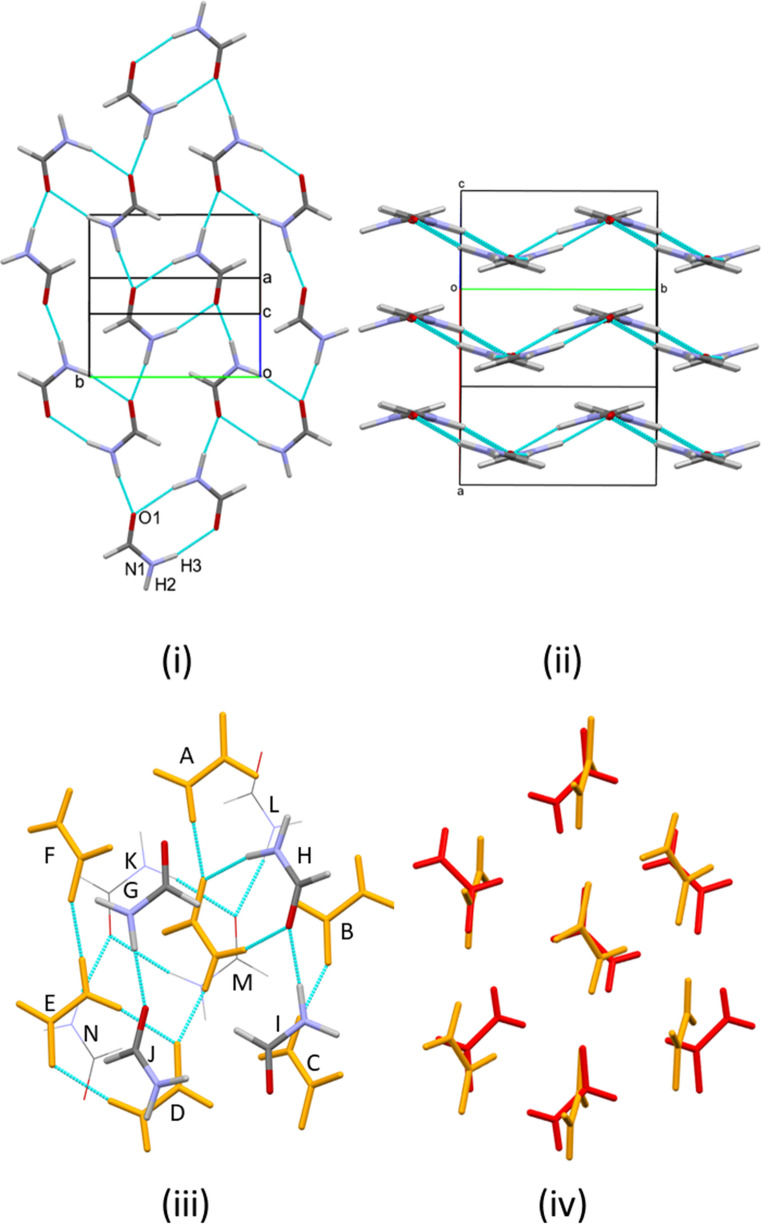
Formamide-III at 0.35 GPa. (i) Formation of H-bonded layers viewed approximately along (-201). (ii) Stacking of the puckered layers. (iii) Coordination sphere, cf. Scheme 2ii. The central layer is in orange. (iv) Fit of the O positions in the central layers of the coordination spheres of phases I_α_ (red) and III (orange)

As in phases I_α_ and II, the structure contains N1H2⋯O1 H-bonded C(4) chains [O1⋯H2 1.819(11) Å]. The angle between the planes of successive molecules along the chain is 26.9°. The acceptor is the O1-based lone pair trans to N1, enabling re-formation of R^2^_2_(8) dimers through inversion-related pairs of N1H3⋯O1 H-bonds [O1⋯H3 2.030(15) Å] which link the chains to form layers (Fig. 5i). There is good agreement between the positions of surrounding donors and acceptors with the full interaction map shown in Fig. S1iii. This description inspires a distinct sense of déjà vu, as it is the same as for formamide-I_α_. The difference is that the layers are much more sinusoidal than those in formamide-I_α_, as shown in Fig. 5ii. The layers are on average parallel to the (− 201) Miller planes.

The phase II-to-III transition could be taken, in essence, to be a re-entrant formation of phase I_α_ but with more puckered layers. A more detailed analysis of the topology suggests an alternative interpretation. Inclusion of only contacts with repulsion energies of greater than zero in the topology analysis (see Experimental Section) yields a coordination sequence of 13-49-110, but inclusion of a small face on the coordination polyhedron (Ω = 0.18% of the full solid angle) corresponding to a centroid-centroid distances of 5.64 Å gives 14 molecules in the first coordination sphere (Fig. 5iii), with a topologically body-centred cubic arrangement with a coordination sequence of 14-50-110 (cf. Scheme 2ii). This is the most common topology for molecular materials,^[25b]^ and it is this description that will be used here.

A central reference molecule is surrounded in a plane by six other molecules arranged in an approximate hexagon (A-F), the oxygen positions conforming closely to those in phase I_α_ (*RMS*_O_ = 0.735 Å, Fig. 5iv). Four molecules now sit above and below the planes to complete the 14-fold coordination environment. The C(4) chains formed through N1H2⋯O1 H-bonds run diametrically across the hexagons from A to D as in phase I_α_. The R^2^_2_(8) dimers formed either side of the C(4) chain no longer both reside within the central plane of the coordination sphere as they do in phase I_α_, but instead one is formed within the plane between positions D and E and the other to molecule H in the plane above. The sinusoidal layers are therefore likely to have been generated by formation of H-bonds between the layers that existed in phase I_α_ after reorientation of the molecules, rather than by a simple puckering mechanism.

The strongest interactions within the hexagonal layers of the coordination sphere are formed between the molecules connected by the N1H2⋯O2 H-bonds (positions A and D, − 27.5 kJ mol^− 1^). OCNH-aldehyde and aldehyde-aldehyde contacts have energies of − 17.0 and − 14.1 kJ mol^− 1^ in positions B and F, respectively. Of the interactions formed above and below the planes, the R^2^_2_(8) dimer is the strongest interaction (− 57.8 kJ mol^− 1^, position H) in the structure, while a symmetry equivalent of the interaction at B is formed below the plane at position K. These account for 6 of the 14 interactions within the molecular coordination sphere, but the others all have energies of magnitude 5 kJ mol^− 1^ or less.

### Formamide-IV_β_

The structure of formamide-IV_β_ is closely related to formamide-I_α_, as can be seen from the unit cell dimensions, Tables 1 and 2. Both the *a* and *c* axes are very similar in the two phases, but the *b* axis has approximately doubled in length in the higher-pressure phase. The space group is *P*2_1_/*n* and *Z*’ = 2. The structure was determined using single-crystal X-ray diffraction at 0.43 GPa and neutron powder diffraction at 0.78 GPa. The parameters from the latter are discussed here.

**Fig. 6:**
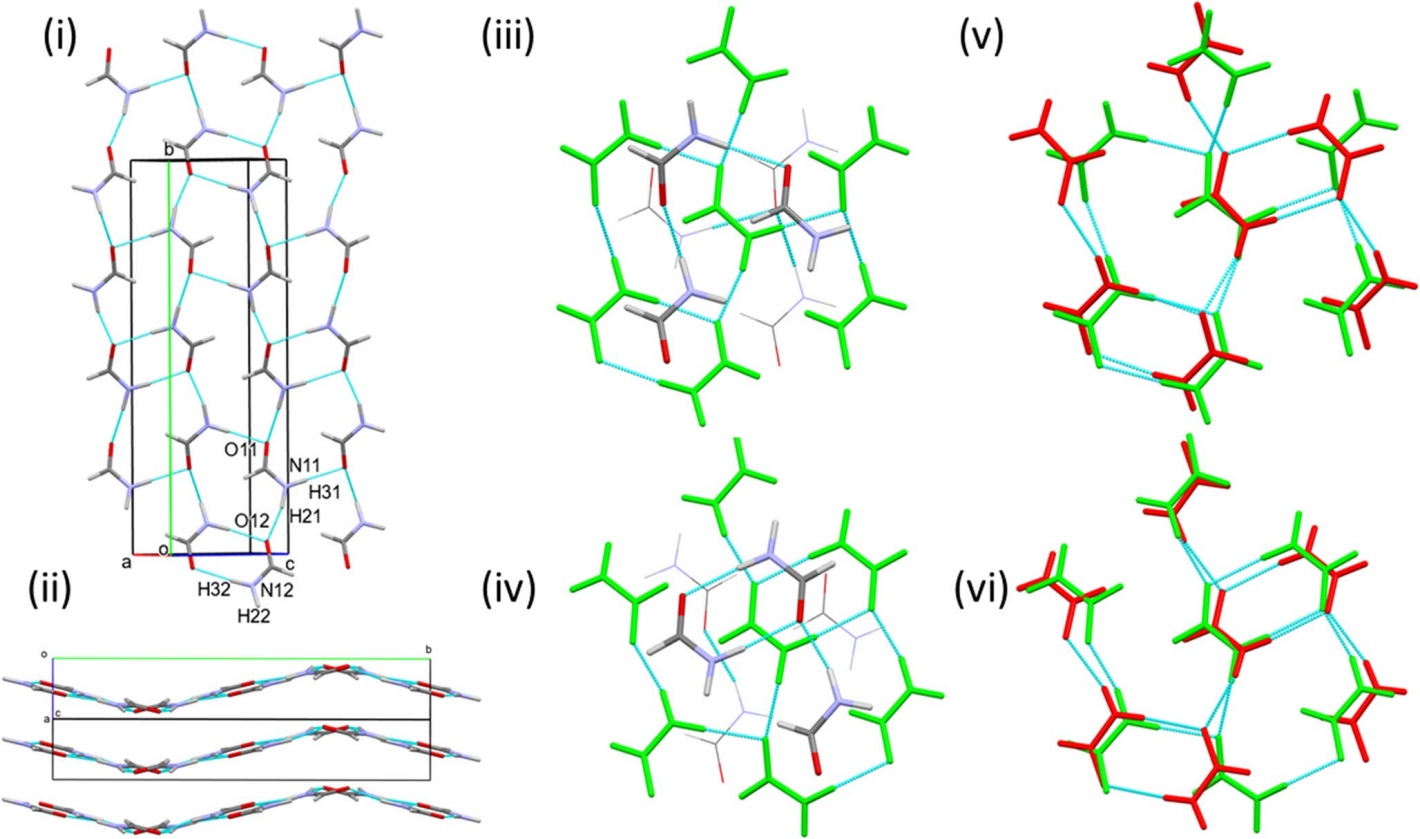
Formamide IV_β_ at 0.78 GPa. (i) Formation of H-bonded layers viewed approximately along (101). (ii) Stacking of the layers. (iii) and (iv) Coordination spheres about molecules 1 and 2, respectively. The central layer is shown in green. (v) and (vi) Fit of the O positions in the central layers of the coordination spheres of phase I_α_ (red) and molecules 1 and 2 in phase IV_β_ (green)

The two crystallographically distinct molecules alternate along chains linked by N11H21⋯O12 and N12H22⋯O11 H-bonds [1.90(2) and 1.834(19) Å, respectively; Fig. 6i]. The motif is very similar to the C(4) chains of phases I-III, but because of the alternation, the formal descriptor is C^2^_2_(8) at secondary level. The chain direction is parallel [010] and the involvement of both crystallographically unique molecules explains why the length of the *b*-axis is approximately double that in phase I_α_. The acceptor oxygen lone pair is cis to the nitrogen atom in molecule 1 (based on O11), but trans in molecule 2, and the alignment with the full interaction map for molecule 1 (Fig. S1iv) is poorer than for molecule 2 (Fig. S1v).

Only molecule 2 is in a suitable configuration to form R^2^_2_(8) rings through inversion-related pairs of N12H32⋯O12 bonds [H32⋯O12 1.97(2) Å]. The remaining N11H31 donor forms a hydrogen bond to the remaining O11 acceptor in a neighbouring chain (see below). The angles between the planes of successive molecules in the chains are 7.1 and 28.2° for the pairs of molecules linked by N11H21⋯O12 and N12H22⋯O11 H-bonds, respectively, and, as in phase I_α_, the combination of H-bonds forms a layer. R^3^_4_(12) rings are formed in addition to the R^2^_2_(8) and R^4^_6_(16) rings that characterised phase I_α_. The layers form parallel to the (101) Miller planes (Fig. 6ii).

The coordination sequence about both unique molecules is 12-42-92, corresponding to the re-adoption of a cubic-close packed topology. Unlike phase III, the H-bonds are contained in the central plane of both coordination spheres, suggesting that formation of phase IV_β_ involves reformation of the layers present in phase I_α_ (Figs. 6iii and iv). The pattern of contacts about molecule 2 is very similar to that in phase I. The energies of the interactions along the C(4) chain being − 27.9 and − 25.8 kJ mol^− 1^ in the A and D positions of the coordination sphere, while that of the R^2^_2_(8) ring is − 61.8 kJ mol^− 1^ in the B position; corresponding figures for these motifs in phase I_α_ are − 30.1 and − 66.1 kJ mol^− 1^, respectively. The layers also feature aldehyde-aldehyde contacts formed at the F position across an inversion centre in which the O⋯H distance is 2.72(3) Å and the energy − 17.1 kJ mol^− 1^. The strongest interactions between the layers are an offset stacking interaction (interplane distance = 3.12 Å) at position H and an aldehyde-aldehyde contact [O12⋯H12 = 2.87(3) Å] at position J with energies of − 14.9 and − 14.5 kJ mol^− 1^, respectively. Other contacts formed are mostly slightly destabilising with energies of up to + 5.1 kJ mol^− 1^. The overlay of the central layers of the coordination spheres of phases I_α_ and IV_β_ (Fig. 6vi) shows a close match (*RMS*_O_ = 0.555 Å), differing only at position D, which is occupied by molecule 1 in the latter.

The pattern of contacts about molecule 1 resembles that in phase II. The molecule-molecule energies along C(4) chain are the same as given above for molecule 2. The two N11H31⋯O11 H-bonds [O11⋯H31 1.972(19) Å] formed either side of the chains at positions B and F in the coordination sphere (as in phase II) are symmetry equivalent and with molecule-molecule energies of − 36.1 kJ mol^− 1^ (− 35.7 kJ mol^− 1^ in phase II). The principal interactions to the layers above and below are a pair of symmetry-equivalent OCNH-aldehyde contacts with O11⋯H11 = 2.62(3) Å. The overlay for the in-plane environments molecule 1 in phase IV_β_ and phase I_α_ shows a larger deviation than for molecule 2 (*RMS*_O_ = 0.958 Å), as expected, but closely resembles the overlay for phase II shown in Fig. 4iv.

In short, the use of both the cis and trans lone pairs as O-based H-bond acceptors in the formation of the ‘C(4)’ chain in phase IV_β_ leads to a structure that can be considered to be a combination of phases I_α_ and II.

**Fig. 7:**
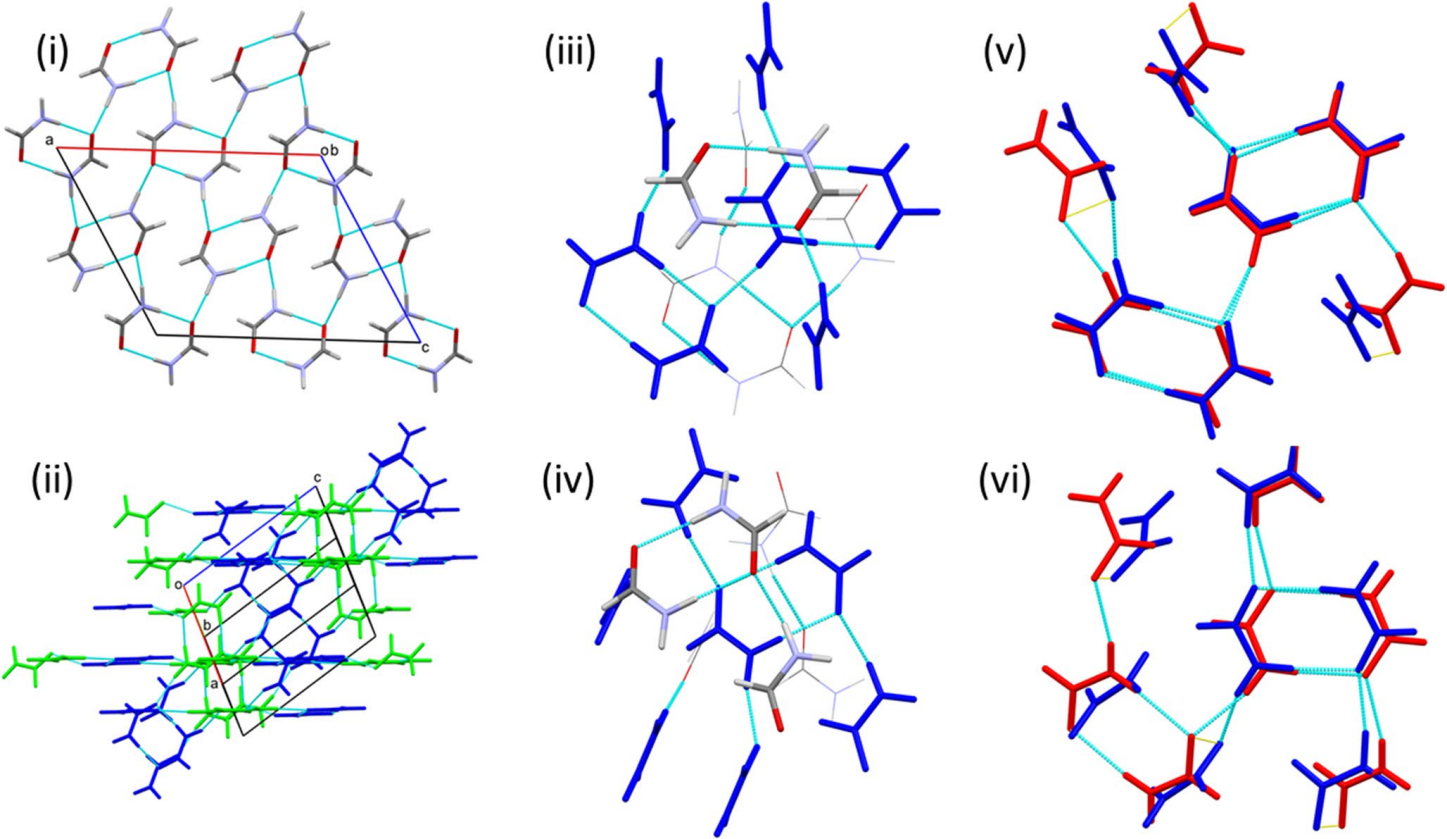
Formamide V at 1.81 GPa. (i) View of the structure projected (i) along and (ii) perpendicular to **b**. Colours in (ii) show crystallographic equivalence. (iii) and (iv) Coordination spheres about molecules 1 and 2, respectively. The central layer is shown in blue. (v) and (vi) Fit of the O positions in the central layers of the coordination spheres of phase I_α_ (red) and molecules 1 and 2 in phase V (blue)

### Formamide-V

The structure of formamide-V is monoclinic, space group *P*2_1_/*a*, with *Z*’ = 2. The structure was determined using neutron powder diffraction at 1.81 GPa.

Like phase I_α_, the structure is based on chains of molecules linked in head-to-tail fashion through NH⋯O H bonds. The two crystallographically distinct molecules alternate along the chain forming N11H21⋯O12 and N12H22⋯O11 H-bonds [O⋯H = 1.891(14) and 1.880(16) Å] with molecule-molecule energies of − 24.8 and − 28.9 kJ mol^− 1^. The formal graph set descriptor is C^2^_2_(8), as in phase IV_β_, but unlike phase IV_β_, the acceptor in both cases is the O-based lone pair trans to the N atom. The H-bond functionality is therefore similar to that in phase I_α_, showing good agreement with full interaction maps shown in Figs. S1vi and vii, forming R^2^_2_(8) dimers formed across inversion centres through N11H31⋯O11 and N12H32⋯O12 (− 61.0 and − 60.4 kJ mol^− 1^) in an alternating pattern either side of the chains (Fig. 7i). Indeed, when projected along **b** the structure of phase V strongly resembles that of phase I_α_. However, in phase I_α_ the molecules were all oriented approximately in the planes of H-bonded layers, but this is not the case in phase V, where the angles between the planes of successive molecules in the N11H21⋯O12 and N12H22⋯O11 chains is 70.3 and 6.6°, respectively. The effect is to generate a 3D network, as in phase II (Fig. 7ii).

The molecular coordination spheres contain 12 molecules in both cases. In the case of the sphere about molecule 1 (Fig. 7iii), both R^2^_2_(8) dimers lie within this plane at position B and between molecule D and E. The molecules not involved in the dimers, at positions A, C and F, are reoriented perpendicular to the plane and form H-bonds to the planes above and below. The reorientations, which in two cases (A and C) occur about directions perpendicular to the C-N bonds, impinge on the plane above, creating space in the plane below so that the coordination sphere is completed by two molecules above and four below the reference plane, rather than three in each as was the case is phase I_α_. The cubic close packed topology of phase I_α_ is disrupted and the coordination sequence changes from 12-42-92 to 12-45-98.

The coordination about molecule 2 is similar, except that only one R^2^_2_(8) ring lies in the reference plane at position B; others are formed to the layer below, but the contacting molecules lie outside the reference coordination sphere. Molecules in the D, E and F positions undergo reorientation by rotating about the C-N bond. This causes less disruption to the planes above and below than was the case for molecule 1. Three molecules are located above and below the reference plane, but are displaced from the positions expected for ideal cubic close packed topology. The coordination sequence is 12-43-99.

In both cases, the larger number of molecules in higher coordination spheres are indicative of a denser packing arrangement. Other significant contacts about molecule 1 take the form of an offset antiparallel stacking interaction to the upper plane (stacking distance = 3.01 Å, − 14.5 kJ mol^− 1^) and two equivalent interactions featuring short CH⋯O distances (H⋯O 2.31(2) Å, − 12.3 kJ mol^− 1^). In the case of molecule 2, aldehyde-aldehyde interactions are formed within the reference plane and to the one below (− 15.3 kJ mol^− 1^), while a short offset stacking interaction (2.54 Å) occurs to the plane above (− 13.4 kJ mol^− 1^).

## Conclusions

The foregoing analysis has depended critically on two seminal advances, which both occurred in the first five years of the present century. Although Kitaigorodski [30] recognised the relationship between hard sphere and molecular structures, it was Peresypkina and Blatov [25] who systematised this type of analysis, using the program Topos to enable precise classification of structure type and analysing the frequencies of different topological types in the Cambridge Database. In the context of the work on formamide described above, the topological approach revealed the close relationship that exists between the different phases.

The second advance was Gavezzotti’s Pixel method.^[22b]^ Prior to this work, the stability of crystal structures was usually analysed in terms of close atom-atom contacts, often using a prehistoric [31] collection of van der Waals radii. One of us (SP) well recalls the revelation of reading Dunitz and Gavezzotti’s paper [32] on interpretation of crystal structures instead in terms of molecule-molecule energies broken down into chemically meaningful electrostatic, polarisation, dispersion and Pauli repulsion contributions. We published our first paper using the method shortly afterwards [33], and it has been a mainstay of our approach to structure interpretation ever since. As we have seen in this work on formamide, many interactions within the first molecular coordination sphere have virtually no influence on the lattice energy so that assigning energies to interactions greatly simplifies structure interpretation by focussing attention on the really significant interactions. It is therefore a great pleasure to dedicate this paper to Professor Gavezzotti.

We have shown here that no less than five phases of formamide can be formed within a window of only 0–2 GPa. The structures of phases II-V were determined by neutron powder diffraction using formamide-*d*_3_, while those of I_α_, II and IV_β_ were determined by single-crystal X-ray diffraction using formamide-*h*_3_. Some differences in the pressures at which phases were observed in the two sets of data and the lack of formation of phase I_α_ in the neutron study may be deuteration effects. It is even possible that phases III and V exist only for formamide-*d*_3_; such a finding would resemble results for pyridine, where a transition from phase I (*Pna*2_1_ and *Z*’ = 4) to phase II (*P*2_1_2_1_2_1_, *Z*’ = 1) is observed in variable-temperature experiments for pyridine-*d*_5_ but not for pyridine-*h*_5_ [34]. None of these potential deuteration effects were investigated systematically in the present study. Neither do our data enable the subtle differences between NH and ND or CH and CD distances expected on the basis of the Ubbelohde effect [35] to be defined, as the neutron data were collected at medium resolution and the structures refined using rigid bodies. Some of these questions could be addressed, for example, by repeating the neutron powder experiment with the Paris-Edinburgh cell using formamide-*h*_3_ or the X-ray experiments with formamide-*d*_3_, but insight from theory, perhaps in the form of periodic density functional theory calculations described by Li et al. [36] would also be extremely valuable.

The formation of so many phases of a molecular material within such a narrow range of pressure is unprecedented. H-bonding is clearly the structure-directing factor in the crystal structures of formamide, and the feature common to all five structures is the NH…O hydrogen-bonded chain. Further NH⋯O hydrogen bonds link the chains to form either layered (I_α_, III and IV_β_) or network (II and V) structures. Which type of structure is formed depends on whether the oxygen acceptor lone pair is cis or trans to the nitrogen atom and on the orientation of the molecules, rather than changes in position.

Full interaction maps (Fig. S1) [29] show that the geometrically most optimal H-bonds are formed when chain formation occurs via the trans lone pairs. Where this is the case, there is a very good match between the positions of H-bonding groups and the distribution expected from entries in the Cambridge Structural Database. The match is much less optimal when the cis lone pair is used (Figs. S1ii and iv), with the position of the donor nitrogen atom of the molecule in the A position of the coordination sphere located between the maxima of the map.

Nevertheless, the molecule-molecule energies for contact A span the range − 24.8 to − 30.1 kJ mol^− 1^ for the bonds involving the trans lone pairs and − 26.7 and − 25.7 kJ mol^− 1^ for those involving the cis lone pairs. The motifs are thus energetically competitive, but which is formed has important consequences for formation of other H-bonds, as only use of the trans lone pair enables formation of R^2^_2_(8) rings.

R^2^_2_(8) rings occur in all phases except phase II and are a common feature of amide structures generally. The molecule-molecule energy is of the order of − 60 kJ mol^− 1^, which is highly stabilising both as a result of the presence of two H-bonds, but also because the short centroid-centroid distance (e.g. 3.704 Å versus 4.745 Å for the C(4) chains in phase I_α_) enhances the contribution of dispersion. Conversely, chain-motif H-bonds have a smaller repulsion term, so that the choice between forming two chain motifs or one ring motif is quite finely balanced.

The angle between the planes of successive molecules along the C(4) chains spans the range 6.6° to 70.3° for the molecules connected by O12⋯H21N12 and O11⋯H22N12, respectively, both in phase V, respectively, but the energies of these interactions mostly fall in the range − 25 to − 30 kJ mol^− 1^. The lack of a strong orientation preference appears to be another driver of polymorphism as it defines the directionality of interactions formed between chains, for example, angles of 54.4° in phase II and 70.25° in phase V are associated with formation of three-dimensional networks.

While the flexibility of H-bond geometry is an important factor in the phase diversity of formamide, all phases show a number of other interactions with molecule-molecule energies between − 10 and − 20 kJ mol^− 1^. These include anti-parallel stacking interactions with distances of between 3.36 and 2.54 Å in phases I_α_ and V, aldehyde-aldehyde interactions with O…H distances of between 2.62 and 2.99 Å in phases I_α_ and III, and interactions between an aldehyde and the OCNH side of another formamide molecule with O…H distances of between 2.62(2) and 2.96(2) Å for molecule 1 in phase IV_β_ and phase II. These contacts, too, show considerable geometric flexibility with little variation in energy.

The theme of the preceding paragraphs has been flexibility arising from which acceptor function is used in H-bonding, the competitive energetics of R and C H-bonding motifs, the relative orientations of molecules forming C-type motifs and in the competitiveness of significant non-H-bonding interactions. The structures of the phases are mostly topologically cubic close packed, exceptions being phase III and phase V, though in the latter case the deviation is only evident beyond the first coordination sphere.

The transitions between phases thus occur with relatively modest changes in the positions of the molecules and instead involve reorientations of some of the molecules present in the first molecular coordination sphere. That these occur so readily may stem from the small size of the formamide molecule and the lack of steric encumbrances to changes in orientation. There is even a degree of consistency in the energies of the interactions within the molecular coordination sphere. Molecules A and D are by definition C-type H-bonds with energies near − 30 kJ mol^− 1^, but the interaction at site B is usually a H-bond (the exception is phase III), while interactions at C, E, I and L are always of negligible importance.

It is presumably the flexibility of the interactions and the topological similarity between the different phases which is responsible for the phase diversity exhibited by formamide.

## Supplementary Information

Below is the link to the electronic supplementary material.

Supplementary Material 1

## Data Availability

Structural and intensity data for all structures reported are available in cif format and have been deposited with the Cambridge Crystallographic Data Centre with deposition numbers 2514855-2514869. The ESI contains Fig. S1 which shows the full interaction maps discussed in the text. Tables S1-S5b show molecule-molecule energies within the first coordination sphere.
