# Supplementary material for: Five phases of formamide formed from 0.1 to 1.8 GPa
Source: Struct Chem. 2026 Mar 30;37(3):1149–64. doi: 10.1007/s11224-026-02744-2 (PMC13179902; doi:10.1007/s11224-026-02744-2)
Supplement: Supplementary file 1 — Supplementary Material 1 [file 11224_2026_2744_MOESM1_ESM.pdf]

SUPPLEMENTARY MATERIAL

Five Phases of Formamide Formed From 0.1 To 1.8 GPa

Alice Dawson,[1] Laura E. Budd,[1] David R. Allan,[1,3] Richard M. Ibberson,[2,4] William G. Marshall[2] and Simon Parsons[1]*

1. EaStCHEM School of Chemistry and Centre for Science at Extreme Conditions, The University of Edinburgh, King's Buildings, West Mains Road, Edinburgh, EH9 3FJ, UK.

2. ISIS Neutron and Muon Source, Rutherford Appleton Laboratory Harwell Campus, Didcot Oxfordshire, OX11 0QX. UK

3. Current address: Diamond House, Harwell Science and Innovation Campus, Didcot, Oxfordshire, OX11 0DE

4. Current address: Neutron Technologies Division, Oak Ridge National Laboratory, 1 Bethel Valley Road, Oak Ridge, TN 37831, USA

Contents

Page 2: Fig S1: Full interaction maps calculated for (i) phase I_{α}, (ii) phase II, (iii) phase III, (iv) molecule 1 in phase IV_{β}, (v) molecule 2 in phase IV_{β}, (vi) molecule 1 in phase V, (vii) molecule 2 in phase V.

Pages 3-9: Tables S1-S5b: Molecule-molecule energies within the first coordination spheres of each phase.

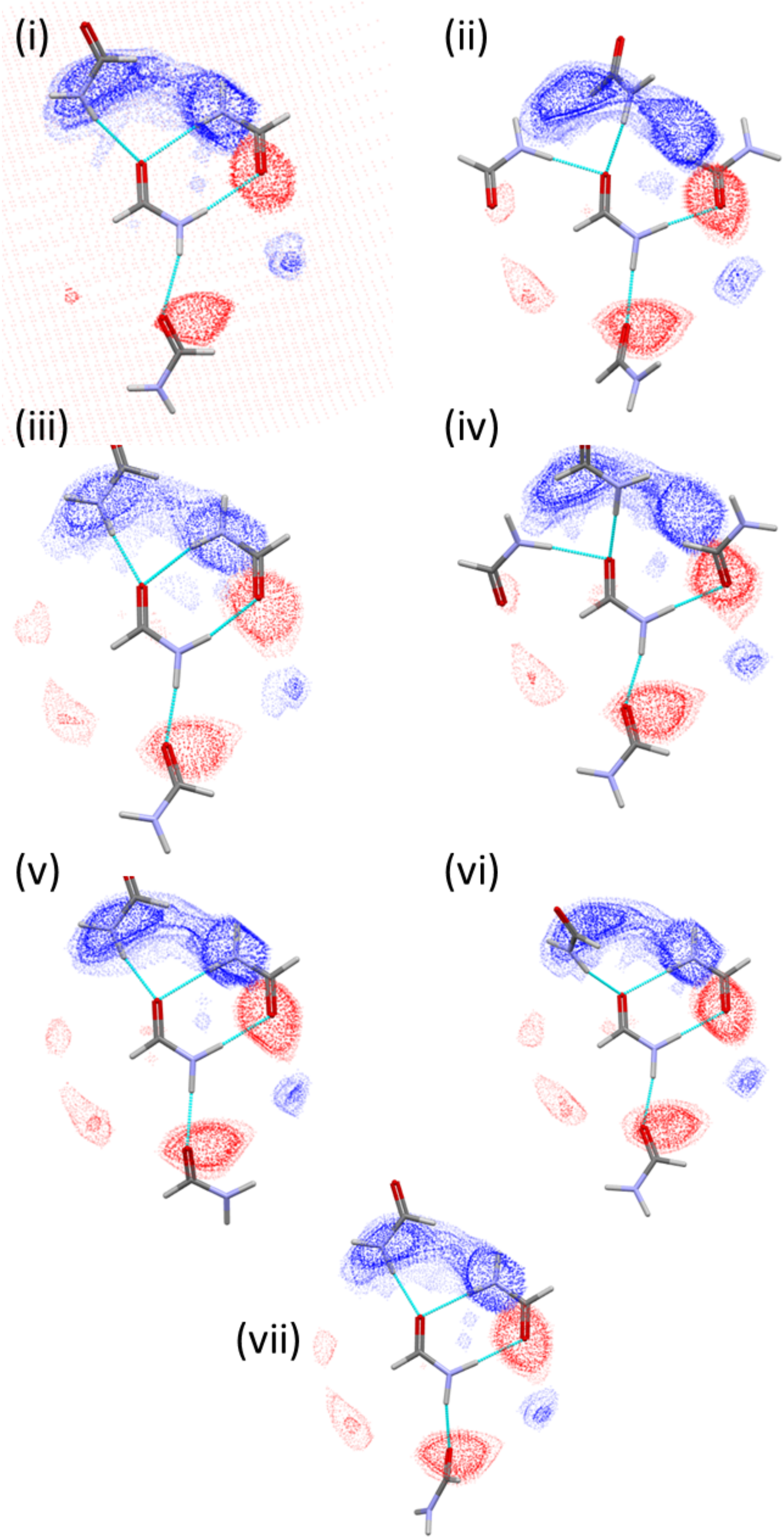

Figure S1: Full interaction maps calculated for (i) phase I_{α}, (ii) phase II, (iii) phase III, (iv) molecule 1 in phase IV_{β}, (v) molecule 2 in phase IV_{β}, (vi) molecule 1 in phase V, (vii) molecule 2 in phase V.

Table S1: Intermolecular interaction energies in the molecular coordination sphere of formamide-I$_\alpha$ at 0.2 GPa. The total energy E_{Tot} is broken down into electrostatic (E_{elec}), polarisation (E_{pol}), dispersion (E_{disp}) and Pauli repulsion (E_{rep}) contributions. All energies were calculated using the Pixel method and are quoted in kJ mol^{-1}. Centroid-centroid distances *d* are given in Å. The same comments apply to Tables S2-5b. For this structure, distances involving H have been 'normalised' to neutron values.

	Symmetry operation	*d*	E_{elec}	E_{pol}	E_{disp}	E_{rep}	E_{tot}	Prominent contacts
A	−*x*+½, *y*−½, −*z*+½	4.745	−41.3	−12.9	−8.1	32.2	−30.1	O1···H2N1 H-bond (O···H = 1.89 Å) in C(4) motif.
B	−*x*+1, −*y*, −*z*	3.704	−81.8	−25.5	−17.0	57.6	−66.6	O1···H3N1 (O···H = 1.94 Å). Part of $R^2_2(8)$ motif
C	*x*+½, −*y*+½, *z*−½	4.887	5.1	−0.9	−3.0	0.7	2.0	Electrostatically destabilising H···H contacts, min distance = 2.84Å.
D	−*x*+½, *y*+½, −*z*+½	4.745	−41.3	−12.9	−8.1	32.2	−30.1	C(4)-building H-bond. Symmetry equivalent of A.
E	*x*−½, −*y*+½, *z*+½	4.887	5.1	−0.9	−3.0	0.7	2.0	Symmetry equivalent of C.
F	−*x*, −*y*, −*z*+1	5.516	−6.5	−0.4	−1.8	0.4	−8.4	Long range electrostatic contact.
G	*x*−1, *y*, *z*	3.681	10.1	−1.3	−5.5	2.3	5.6	Off-set interlayer stacking interaction. Dipoles parallel.
H	−*x*, −*y*, −*z*	3.852	−9.9	−1.3	−4.6	1.3	−14.5	Off-set interlayer stacking interaction (stacking distance = 3.36 Å, O···H 3.37 Å). Dipoles opposed.
I	*x*−½, −*y*+½, *z*−½	4.447	−0.3	−0.9	−3.4	0.9	−3.8	Non-specific interlayer dispersion interaction.
J	−*x*+1, −*y*, −*z*+1	4.592	−15.6	−3.4	−6.9	9.1	−16.9	Aldehyde-aldehyde interaction. O···H = 2.62 Å
K	*x*+1, *y*, *z*	3.681	10.1	−1.3	−5.5	2.3	5.6	Symmetry equivalent of G.
L	*x*+½, −*y*+½, *z*+½	4.447	−0.3	−0.9	−3.4	0.9	−3.8	Symmetry equivalent of I.

Table S2: Intermolecular interaction energies in the molecular coordination sphere of formamide-II at 0.2 GPa..

	Symmetry operation	d	E_{elec}	E_{pol}	E_{disp}	E_{rep}	E_{tot}	Prominent contacts
A	x−½, −y+½, z	4.766	−37.6	−11.8	−7.7	30.4	−26.7	O1···H2N1 [O···H = 1.90(2) Å] in C(4) chain
B	−x+³⁄₂, y+½, z+½	4.279	−47.8	−16.0	−11.9	40.1	−35.7	N1H3···O1 [O···H = 1.881(18) Å, graph set descriptor also C(4)]
C	−x+2, −y+1, z+½	4.733	5.6	−1.0	−3.0	0.5	2.1	Destabilising H···H contacts. Shortest H···H = 2.70(2) Å
D	x+½, −y+½, z	4.766	−37.6	−11.8	−7.7	30.4	−26.7	Symmetry equivalent of A
E	−x+2, −y, z−½	4.404	−1.6	−1.3	−4.5	3.2	−4.2	Non-specific dispersion interaction.
F	−x+³⁄₂, y−½, z−½	4.279	−47.8	−16.0	−11.9	40.1	−35.7	Symmetry equivalent of B
G	x, y−1, z	3.751	8.9	−1.1	−5.3	2.7	5.2	Off-set interlayer stacking interaction. Dipoles parallel.
H	−x+³⁄₂, y−½, z+½	4.279	−8.3	−1.5	−4.0	1.5	−12.3	Electrostatic interaction involving OCNH···aldehyde min O1···H1 = 2.96(2) Å.
I	−x+2, −y, z+½	4.404	−1.6	−1.3	−4.5	3.2	−4.2	Symmetry equivalent of E
J	−x+³⁄₂, y+½, z−½	4.279	−8.3	−1.5	−4.0	1.5	−12.3	Symmetry equivalent of H
K	x, y+1, z	3.751	8.9	−1.1	−5.3	2.7	5.2	Symmetry equivalent of G
L	−x+2, −y+1, z−½	4.733	5.6	−1.0	−3.0	0.5	2.1	Symmetry equivalent of C

Table S3: Intermolecular interaction energies in the molecular coordination sphere of formamide-III at 0.35 GPa..

	Symmetry operation	*d*	E_{elec}	E_{pol}	E_{disp}	E_{rep}	E_{tot}	Prominent contacts
A	*x*+½, −*y*+³⁄₂, *z*+1	4.877	−46.4	−15.8	−8.5	43.1	−27.5	O1···H2N1 [O···H = 1.819(11) Å] in C(4) chain
B	−*x*+³⁄₂, *y*+½, −*z*−1	4.245	−12.5	−2.0	−6.4	4.0	−17.0	Electrostatic interaction involving OCNH···aldehyde. Shortest distance is O1···H1 = 2.86(2) Å.
C	−*x*+1, −*y*+2, −*z*−2	4.792	2.4	−0.7	−2.8	0.6	−0.6	Neutral interaction where small destabilising electrostatic and stabilising dispersion terms effectively cancel.
D	*x*−½, −*y*+³⁄₂, *z*−1	4.877	−46.4	−15.8	−8.5	43.2	−27.5	Symmetry equivalent of A
E	−*x*+³⁄₂, *y*−½, −*z*−2	4.473	4.7	−1.5	−4.7	1.8	0.3	Neutral interaction where small destabilising electrostatic and stabilising dispersion terms effectively cancel.
F	−*x*+2, −*y*+1, −*z*−1	4.816	−10.4	−1.6	−4.1	2.1	−14.1	Aldehyde-aldehyde interaction O···H = 2.99(2) Å.
G	*x*+½, −*y*+³⁄₂, *z*	3.529	10.0	−1.7	−6.8	3.9	5.5	Destabilising inter-carbonyl interaction C···C and O···O = 3.48 and 3.47 Å. Offset by non-specific dispersion.
H	−*x*+2, −*y*+2, −*z*−1	3.794	−64.9	−17.5	14.3	38.9	−57.8	$R^2_2(8)$ H-bonded dimer formed through N1H3···O1 [H···O = 2.030(15) Å]
I	−*x*+³⁄₂, *y*+½, −*z*−2	4.473	4.7	−1.5	−4.7	1.8	0.3	Symmetry equivalent of E
J	*x*, *y*, *z*+1	5.302	−0.2	−0.6	−1.2	0.1	−1.8	Long-range, energetically neutral interaction
K	−*x*+³⁄₂, *y*−½, −*z*−1	4.245	−12.5	−2.0	−6.4	4.0	−17.0	Symmetry equivalent of B
L	*x*, *y*, *z*−1	5.302	−0.2	−0.6	−1.2	0.1	−1.8	Long-range, energetically neutral interaction
M	*x*−½, −*y*+³⁄₂, *z*	3.529	10.0	−1.7	−6.8	3.9	5.5	Symmetry equivalent of G
N	−*x*+1, −*y*+1, −*z*−2	5.636	1.0	−0.2	−0.6	0.0	0.1	Long-range, energetically neutral interaction.

Table S4a: Intermolecular interaction energies in the molecular coordination sphere of molecule 1 in formamide-IV$_{\beta}$ at 0.78 GPa. The notation A(2) means that a symmetry equivalent of molecule 2 occupies position A in the coordination sphere.

	Symmetry operation	*d*	E_{elec}	E_{pol}	E_{disp}	E_{rep}	E_{tot}	Prominent contacts
A(2)	*x*−½, −*y*+½, *z*−½	4.772	−36.5	−11.5	−7.7	30.0	−25.7	O11···H22N12 [O···H = 1.90(2) Å] in $C^2_2(8)$ chain
B(1)	*x*−½, −*y*+½, *z*+½	4.242	−41.8	−12.7	−11.5	30.0	−36.0	N11H31···O11 [O···H = 1.972(19) Å] H-bond
C(2)	*x*−1, *y* , *z*	4.628	6.5	−1.7	−4.7	2.3	2.5	Destabilising H···H contacts. Shortest H···H = 2.41(2) Å
D(2)	−*x*, −*y*, −*z*+2	4.823	−45.8	−15.2	−8.9	41.9	−27.9	N11H21···O12 [O···H = 1.834(19) Å] in $C^2_2(8)$ chain
E(2)	*x*, *y*, *z*−1	4.616	5.4	−1.8	−5.1	3.1	1.5	Destabilising H···H contacts. Shortest H···H = 2.442(18) Å
F(1)	*x*+½, −*y*+½, *z*−½	4.242	−41.8	−12.7	−11.5	29.9	−36.1	Symmetry equivalent of B
G(1)	*x*−½, −*y*+½, *z*−½	4.070	−12.6	−2.6	−6.2	5.7	−15.7	Electrostatic interaction involving OCNH…aldehyde. Shortest O11···H11 = 2.62(3) Å
H(1)	*x*−1, *y*, *z*	3.585	9.5	−1.4	−6.6	4.6	6.1	Off-set interlayer stacking interaction. Dipoles parallel
I(2)	*x*−1, *y*, *z*−1	4.914	1.3	−0.5	−1.6	0.1	−0.7	Non-specific, energetically neutral interaction.
J(1)	*x*+1, *y*, *z*	3.585	9.5	−1.4	−6.6	4.6	6.1	Symmetry equivalent of H
K(1)	*x*+½, −*y*+½, *z*+½	4.070	−12.6	−2.6	−6.2	5.7	−15.7	Symmetry equivalent of G
L(2)	*x*, *y*, *z*	3.963	−2.7	−1.3	−7.7	3.1	−6.8	Offset interlayer stacking interaction

Table S4b: Intermolecular interaction energies in the molecular coordination sphere of molecule 2 in formamide-IV$_{\beta}$ at 0.78 GPa.

	Symmetry operation	*d*	E_{elec}	E_{pol}	E_{disp}	E_{rep}	E_{tot}	Prominent contacts
A(1)	−*x* , −*y*, −*z*+2	4.823	−45.8	−15.2	−8.9	41.9	−27.9	O12···H21N11 [O···H = 1.834(19) Å] in $C^2_2(8)$ chain
B(2)	−*x*, −*y*, −*z*+3	3.702	−74.1	−22.4	−16.3	50.9	−61.8	O12···H32N12 [O···H = 1.97(2) Å] in $R^2_2(8)$ dimer
C(1)	*x*, *y*, *z*+1	4.616	5.4	−1.8	−5.1	3.1	1.5	Destabilising H···H contacts. Shortest H···H = 2.442(18) Å
D(1)	*x*+½, −*y*+½, *z*+½	4.772	−36.5	−11.5	−7.7	30.0	−25.8	N12H12···O11 [O···H = 1.90(2) Å] in $C^2_2(8)$ chain
E(1)	*x*+1, *y*, *z*	4.628	6.5	−1.7	−4.7	2.3	2.5	Destabilising H···H contacts. Shortest H···H = 2.41(2) Å
F(2)	−*x*+1, −*y*, −*z*+2	4.788	−14.4	−3.1	−7.0	7.4	−17.1	Aldehyde-aldehyde contact. O···H = 2.72(3) Å
G(2)	*x*+1, *y* , *z*	3.585	9.7	−1.5	−6.8	3.7	5.1	Off-set interlayer stacking interaction. Dipoles parallel
H(2)	−*x*+1, −*y*, −*z*+3	3.625	−10.5	−1.9	−6.7	4.2	−14.9	Off-set interlayer stacking interaction. Dipoles opposed. Stacking distance = 3.12 Å
I(1)	*x*+1, *y*, *z*+1	4.914	1.3	−0.5	−1.6	0.1	−0.7	Non-specific, energetically neutral interaction.
J(2)	−*x*, −*y*, −*z*+2	4.542	−11.4	−2.7	−6.0	5.6	−14.5	Aldehyde-aldehyde contact [O···H = 2.87(3) Å]
K(2)	*x*−1, *y*, *z*	3.585	9.7	−1.5	−6.8	3.7	5.1	Symmetry equivalent of G
L(1)	*x*, *y*, *z*	3.963	−2.7	−1.3	−5.9	3.1	−6.8	Offset interlayer stacking interaction

Table S5a: Intermolecular interaction energies in the molecular coordination sphere of molecule 1 in formamide-V at 1.81 GPa.

	Symmetry operation	*d*	E_{elec}	E_{pol}	E_{disp}	E_{rep}	E_{tot}	Prominent contacts
A(2)	*x*+½, −*y*−½, *z*+1	4.660	−39.9	−14.2	−9.1	38.4	−24.8	O11···H22N12 [O···H = 1.880(16) Å] in $C^2_2(8)$ chain
B(1)	−*x*−1, −*y*−2, −*z*	3.624	−80.0	−26.1	−17.7	62.8	−61.0	O11···H31N11 [O···H = 1.928(18) Å] in $R^2_2(8)$ dimer
C(2)	−*x*−³⁄₂, *y*−½, −*z*−1	4.354	3.8	−2.6	−6.7	5.5	0.1	Energetically neutral contact featuring destabilising H···H contacts. Shortest H···H = 2.306(16) Å
D(2)	*x*, *y*, *z*	4.920	−40.6	−12.6	−7.8	32.1	−28.9	N11H21···O12 [O···H = 1.891(14) Å] in $C^2_2(8)$ chain
E(2)	−*x*−1, −*y*, −*z*−1	4.763	5.9	−1.2	−3.9	1.3	2.0	Destabilising H···H contacts. Shortest H···H = 2.591(16) Å
F(1)	−*x*−½, *y*+½, −*z*	4.685	−14.6	−4.1	−6.6	13.0	−12.3	CH···O contact H11···O11 = 2.31(2) Å
G(1)	*x*, *y*+1, *z*	3.469	8.0	−2.6	−9.2	8.0	4.2	Off-set interlayer stacking interaction. Dipoles parallel
H(1)	−*x*−1, −*y*−1, −*z*	3.352	−12.2	−2.5	−9.5	9.7	−14.5	Off-set interlayer stacking interaction. Dipoles opposed. Stacking distance = 3.01 Å
K(2)	−*x*−1, −*y*−1, −*z*−1	3.774	−5.5	−1.8	−7.8	6.6	−8.6	Off-set interlayer stacking interaction. Shortest N···H = 2.937(14) Å.
L(1)	−*x*−½, *y*−½, −*z*	4.685	−14.6	−4.1	−6.6	13.0	−12.3	Symmetry equivalent of F
M(1)	*x*, *y*−1, *z*	3.469	8.0	−2.6	−9.2	8.0	4.2	Symmetry equivalent of G
N(2)	*x*, *y*−1, *z*	4.745	−2.4	−1.5	−3.3	2.1	−5.1	Off-set interlayer stacking interaction. Shortest H···O = 2.954(17) Å.

Table S5b: Intermolecular interaction energies in the molecular coordination sphere of molecule 2 in formamide-V at 1.18 GPa.

	Symmetry operation	*d*	E_{elec}	E_{pol}	E_{disp}	E_{rep}	E_{tot}	Prominent contacts
A(2)	*x*, *y*, *z*	4.920	−40.6	−12.6	−7.8	32.1	−28.9	O12···H21N21 [O···H = 1.891(14) Å] in $C^2_2(8)$ chain
B(2)	−*x*−1, −*y*, −*z*−1	3.647	−75.7	−24.6	−17.3	57.2	−60.4	O12···H32N12 [O···H = 1.949(19) Å] in $R^2_2(8)$ dimer
C(1)	−*x*−1, −*y*, −*z*−1	4.763	5.9	−1.2	−3.9	1.3	2.0	Destabilising H···H contacts. Shortest H···H = 2.591(16) Å
D(1)	*x*−½, −*y*−½, *z*−1	4.660	−39.9	−14.2	−9.1	38.5	−24.8	N12H22···O11 [O···H = 1.880(16) Å] in $C^2_2(8)$ chain
E(1)	−*x*−³⁄₂, *y*+½, −*z*−1	4.354	3.8	−2.6	−6.7	5.5	0.1	Energetically neutral contact featuring destabilising H···H contacts. Shortest H···H = 2.306(16) Å
F(2)	−*x*−³⁄₂, *y*−½, −*z*−1	4.344	−12.8	−2.6	−6.7	6.8	−15.3	Aldehyde-aldehyde interaction; molecules perpendicular. [O···H both ~ 2.80(2) Å]
G(2)	*x*, *y*−1, *z*	3.469	6.7	−2.7	−9.8	13.6	7.8	Off-set interlayer stacking interaction. Dipoles parallel
H(2)	−*x*−1, −*y*−1, −*z*−1	4.310	−8.4	−2.5	−4.9	2.4	−13.4	Off-set interlayer stacking interaction. Dipoles opposed. Stacking distance = 2.54 Å.
I(1)	−*x*−1, −*y*−1, −*z*−1	3.774	−5.5	−1.8	−7.8	6.6	−8.6	Off-set interlayer stacking interaction. Shortest N···H = 2.937(14) Å.
J(2)	−*x*−³⁄₂, *y*+½, −*z*−1	4.344	−12.8	−2.6	−6.7	6.8	−15.3	Symmetry equivalent of F
K(1)	*x*, *y*+1, *z*	4.745	−2.4	−1.5	−3.3	2.1	−5.1	Off-set interlayer stacking interaction. Shortest H···O = 2.954(17) Å.
L(2)	*x*, *y*+1, *z*	3.469	6.7	−2.7	−9.8	13.6	7.8	Symmetry equivalent of G
